# Genetic and histological studies on the delayed systemic movement of *Tobacco Mosaic Virus *in *Arabidopsis thaliana*

**DOI:** 10.1186/1471-2156-9-59

**Published:** 2008-09-26

**Authors:** Carolina Serrano, Javiera González-Cruz, Francisca Jauregui, Consuelo Medina, Pablo Mancilla, José Tomás Matus, Patricio Arce-Johnson

**Affiliations:** 1 Departamento de Genética Molecular y Microbiología, Pontificia Universidad Católica de Chile, Casilla 114-D. Santiago, Chile

## Abstract

**Background:**

Viral infections and their spread throughout a plant require numerous interactions between the host and the virus. While new functions of viral proteins involved in these processes have been revealed, current knowledge of host factors involved in the spread of a viral infection is still insufficient. In *Arabidopsis thaliana*, different ecotypes present varying susceptibilities to *Tobacco mosaic virus *strain U1 (TMV-U1). The rate of TMV-U1 systemic movement is delayed in ecotype Col-0 when compared with other 13 ecotypes.

We followed viral movement through vascular tissue in Col-0 plants by electronic microscopy studies. In addition, the delay in systemic movement of TMV-U1 was genetically studied.

**Results:**

TMV-U1 reaches apical leaves only after 18 days post rosette inoculation (dpi) in Col-0, whereas it is detected at 9 dpi in the Uk-4 ecotype. Genetic crosses between Col-0 and Uk-4 ecotypes, followed by analysis of viral movement in F_1 _and F_2 _populations, revealed that this delayed movement correlates with a recessive, monogenic and nuclear locus. The use of selected polymorphic markers showed that this locus, denoted *DSTM1 *(Delayed Systemic Tobamovirus Movement 1), is positioned on the large arm of chromosome II. Electron microscopy studies following the virion's route in stems of Col-0 infected plants showed the presence of curved structures, instead of the typical rigid rods of TMV-U1. This was not observed in the case of TMV-U1 infection in Uk-4, where the observed virions have the typical rigid rod morphology.

**Conclusion:**

The presence of defectively assembled virions observed by electron microscopy in vascular tissue of Col-0 infected plants correlates with a recessive delayed systemic movement trait of TMV-U1 in this ecotype.

## Background

Systemic viral infections in plants are complex processes that require compatible virus-host interactions in multiple tissues. These interactions include: viral genome replication in the cytoplasm of the initially infected cells, cell-to-cell movement towards neighboring tissues, long-distance movement through the vascular tissue, phloem unloading and cell-to-cell movement in non-inoculated systemic tissues [1]. Incompatibilities between virus and host factors at any of these stages could therefore lead to restrictions and delays establishment of a systemic infection.

The *Tobacco mosaic virus *TMV-U1 has been one of the most useful viruses for elucidating the steps of viral infections in experimental plant systems [2,3]. The TMV genome encodes four proteins which participate in several viral functions required for a successful infection. Recent studies have shown that replication and movement of viral complexes in infected tobacco tissues are strongly associated with plant structures such as the endoplasmic reticulum and the cytoskeleton [4-6].

Viral infections in plants have been studied in the model plant *Arabidopsis thaliana*, due to the genetic and genomic knowledge of this specie. This model has proven to be useful in elucidating the relationship between the host plant and both the virus replication and movement processes [7,8]. Several *Arabidopsis *ecotypes display differential susceptibilities towards specific viral infections. This has led to the identification of various loci involved in development of viral infections. For example, some host loci responsible for resistance against viral infections have been located in this model [9-11]. Among these, different genes related to the cell cycle [12,13] and viral movement have been identified [14,15]. Nevertheless, the relationship between host proteins encoded by these genes and viral factors involved in these interactions are still an active research issue [13].

In previous works, we evaluated the systemic infection of TMV-U1 in fourteen ecotypes of *Arabidopsis thaliana *using *in vitro *grown plants [16]. Important differences in the rate of the systemic infection were found among these ecotypes; some, such as Uk-4 became infected at a very fast rate, while others, for example Col-0, became infected very slowly. With the aim of studying this natural variance of *Arabidopsis *ecotypes, we searched for the genetic basis that could explain the differences in viral systemic infection rates in *Arabidopsis thaliana*. For this purpose Uk-4 and Col-0 ecotypes were selected. Genetic crosses were performed between plants of both ecotypes and the resulting progeny was analysed with genetic markers to localize the trait conferring this delay within Col-0. Electron microscopy was employed to identify the tissues in which the virus spread was delayed.

## Methods

### Plant growing and genetic crosses

*Arabidopsis thaliana *ecotypes Columbia-0 (Col-0) and Umkirch-4 (Uk-4) were grown in soil in a controlled environment growth chamber. Col-0 and Uk-4 crosses were carried out according to the method described by Guzmán and Ecker [17] to obtain the F_1 _progeny. Crosses (♀)Uk-4 × (♂)Col-0 and reciprocal crosses (♀)Col-0 × (♂)Uk-4 were also performed. F_1 _plants derived from these crosses were grown in soil under controlled conditions and self- pollinated to obtain F_2 _populations.

### Virus stocks and plant inoculation

Virus stocks of TMV-U1 and TMV-Cg were prepared from systemically infected *Nicotiana tabacum *cvXanthi nn plants as described by Bruening *et al*. [18], and stored in TE-virus buffer (Tris-HCl 10 mM, EDTA 1 mM pH 7.2) at 4°C.

*Arabidopsis thaliana *ecotypes Col-0 and Uk-4, as well as F_1 _and F_2 _plants were grown *in vitro *on half MS solid medium in a climate chamber [25°C, 16/8-h (light/dark) photoperiod]. Six-week-old plants with inflorescences were mechanically inoculated on three rosette leaves with a 10 μg/ml virus solution in 20 mM phosphate buffer pH 7.0, using a sterile cotton swab dusted with carborundum.

### Immunological detection of TMV-U1 in infected plants

Virus presence in plant tissues was detected by Western blot and ELISA using a virus coat protein (CP) antibody [19]. For Western blot experiments, both inoculated and apical leaves of parents, F_1 _and F_2 _populations were harvested at 12 dpi. Fresh *Arabidopsis *leaves (50 mg) were macerated with 100 μl of extraction buffer (125 mM Tris-HCl pH 6.8, 0.1% SDS and 20% v/v glycerol), debris was removed by centrifugation at 10.000 g for 10 minutes and 30 μg of total protein were denatured at 95°C, and separated by 12% polyacrylamide-SDS gel electrophoresis. Proteins were transferred to a nitrocellulose membrane (0,45 μM, Amersham Life Science). Blots were blocked with skim milk and then incubated with a rabbit polyclonal antibody against TMV-U1 CP diluted at 1:1000, as previously described by Arce-Johnson *et al*. [16]. Finally, CP was revealed using conjugated alkaline phosphatase-goat anti-rabbit IgG (ImmunoPure Antibody, Pierce, USA) diluted to 1:20.000 and alkaline phosphatase activity was revealed with "Phosphatase substrate 3-C" (KPL, Maryland, USA).

ELISA assays were performed as described by Pereda *et al*. [19]. Pools of apical leaves from eight plants were homogenized in carbonate buffer at pH 9.6, and total proteins were measured by BCA protein assay reagent A (Pierce, Rockford, USA). Plates were incubated overnight with 100 μg of leaf extract supernatants. Primary antibodies [16] and alkaline phoshatase conjugate (goat anti-rabbit IgG, Pierce, USA) were diluted to 1:500. The enzymatic reaction was incubated for 60 min at 37°C, using 1 mg/mL of 4-paranitrophenyl-phosphate (Merck) as a substrate. Optical density was measured at λ = 405 nm in an ELISA microwell reader (Metertech Σ960, Austria). Relative CP was obtained as the ratio of the absorbance of each sample to the maximum absorbance in the plate corresponding to an inoculated leaf at 10 dpi sample.

### Leaf skeleton hybridization

Inoculated and apical *Arabidopsis *leaves were collected at 10 dpi and prepared for hybridisation as described by Lartey *et al*. [20]. Briefly, chlorophyll was removed from leaves using 95% ethanol and proteins were digested with proteinase K at 45°C for 3 hours. Leaves were incubated in a prehybridisation solution consisting of 6× SSPE, 50% formamide, 5× Denhardt's solution, 0.5% SDS and 20 ug/ml salmon sperm DNA at 42°C for 2 h. A denatured ^32^P labeled probe was added and kept overnight. This probe consisted of a PCR product of 518 bp corresponding to the TMV-U1 *CP *gene. Leaves were washed twice at 42°C in 2.5× SSC, rinsed in distilled water, carefully fixed to a nitrocellulose membrane and then autoradiographed. Three independent experiments were carried out inoculating four plants of each ecotype. Mock inoculated leaves were also used in hybridization experiments as negative controls.

### Electron Microscopy

Inoculated leaves, petioles, stems and apical leaves from non-infected and infected plants were prepared for electron microscopy. Tissue samples of 0.5 cm were taken from plants after 10, 12 and 18 dpi. Tissues were fixed in 0.1 M sodium cacodylate-buffer (pH 7.2) with 3% glutaraldehyde for 3 h at room temperature according to Lartey et al. [20]. Samples were washed in 0.1 M cacodylate buffer for 1 h, dehydrated in an acetone gradient (50, 70, 95 and 100%) and embedded in a freshly prepared Embed 812 (EM Sciences, Fort Washington, USA). After overnight polymerization at 60°C, thin sections (70–80 nm) were cut using a Sorvall MT2-B ultramicrotome. Samples were stained in methanol 4% uranyl acetate [21] followed by 0.1% lead citrate [22] and examined under a Philips Model Tecnai 12 transmission electron microscope. Images were revealed on 6.5 × 9.0 Kodak SO163 film.

### Genetic Analysis

Polymorphisms among the parental ecotypes Col-0 and Uk-4 were studied testing 79 cleaved amplified polymorphic sequences (CAPS) and 53 simple sequence length polymorphisms (SSLP) (Research Genetic, Hunsville, USA), covering all five *Arabidopsis *chromosomes. Information on the CAPS and SSLP markers were obtained from The Arabidopsis Information Resource (TAIR; ) and The Bio-Array Resource for Arabidopsis Functional Genomics (BAR; ). Other markers were also obtained from Muramoto *et al*., [23] and Matsumoto and Okada [24]. Genomic DNA was prepared and small scale PCRs were carried out according to Konieczny and Ausubel [25] for CAPS and according to Bell and Ecker [26] for SSLP. Markers that were polymorphic among both ecotypes were selected for mapping. Mapping was done using the individuals of the F_2 _population which showed delay in TMV-U1 systemic movement detected by Western blot at 12 dpi. Initially, five polymorphic molecular markers for each of the five *Arabidopsis *chromosomes were used. Recombination frequency between the locus and the molecular marker was calculated by dividing the number of recombinant chromatids by the total number of chromatids. For chromosome II, 25 additional polymorphic markers were used in order to map the locus with a higher resolution.

## Results

### Differential time-course of TMV-U1 systemic infection in Col-0 and Uk-4 ecotypes

**Figure 1:**
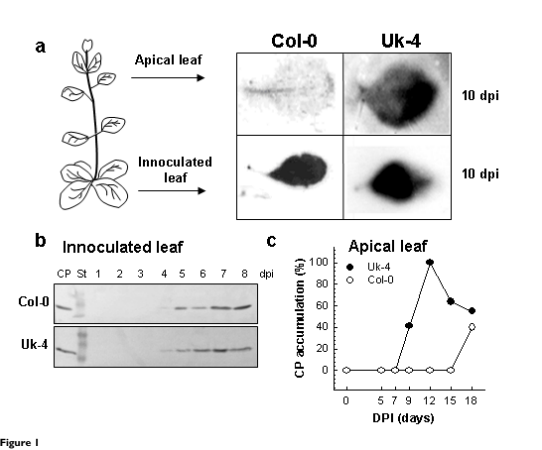
**Differential systemic movement of TMV-U1 in two *Arabidopsis thaliana *ecotypes**. Col-0 and Uk-4 plants were mechanically inoculated on three rosette leaves with a solution of 10 ng/μl of virus diluted in 20 mM phosphate buffer. (a) *In situ *hybridisation of inoculated and apical leaves from infected plants at 10 dpi. Leaves were incubated with a TMV-U1 coat protein DNA probe. The virus coat protein gene was detected ubiquitously in inoculated leaves from both ecotypes, but only in an apical leaf of Uk-4 plants. Images represent equivalent levels of exposure to film. (b) Western blot showing similar time-courses of TMV-U1 infection in inoculated leaves of Col-0 (upper) and Uk-4 (bottom) plants. Coat protein is evident in both cases starting from 4 dpi. (c) ELISA showing differences in the systemic spread of TMV-U1 in Col-0 and Uk-4 plants. Apical leaves of inoculated plants were analysed for 18 dpi.

*In situ *hybridization was used to compare virus accumulation in inoculated and systemic leaves of *Arabidopsis *ecotypes Uk-4 and Col-0. As expected, viral RNA was present in apical leaves of Uk-4 at 10 dpi, but not in those from the Col-0 ecotype (Figure 1a) even when inoculated leaves of both ecotypes were completely infected at this time point (Figure 1a). This result confirms the Uk-4 and Col-0 differences in viral systemic movement as was previously described by Arce-Johnson *et al*. 2000 [16].

To discard that delayed systemic movement in Col-0 was due to a difference in local virus movement, we monitored the time course of the infection in the inoculated leaf for both ecotypes. TMV-CP was detected by Western blot in the inoculated basal leaves starting from 4 dpi in both ecotypes, indicating that the local movement of the virus is not affected by the genetic background (Figure 1b).

Viral systemic movement was further studied by ELISA (Figure 1c). In apical leaves of Uk-4, TMV CP is present starting from 9 dpi, while in Col-0, CP is detectable in these leaves only after 18 dpi. Based on these results, we decided to analyse genetic experimental populations in order to distinguish delayed and susceptible phenotypes in the next steps at 12 dpi, a time point in which the differences in viral CP accumulation in distal apical leaves was clearly evident between Uk-4 and Col-0 ecotypes.

### Curved particles of TMV-U1 appear in the vascular stem tissues in Col-0

**Figure 2:**
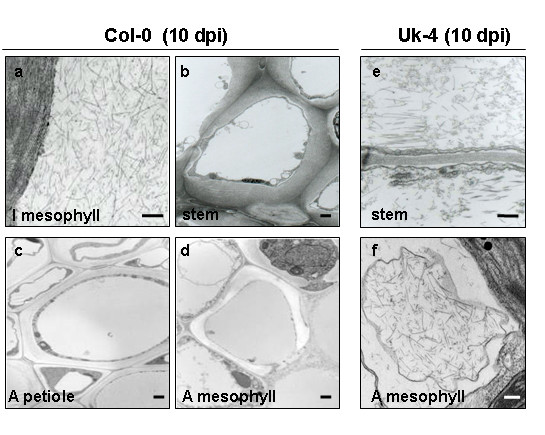
**TMV-U1 particles appear in the inoculated leaves but not in the stems or apical tissues of Col-0 at 10 dpi**. Electron microscopy images showing high accumulation of virions in mesophyll cells of Col-0 inoculated leaves (a), and absence of these particles in vascular tissue of the stem (b), petiole (c) and mesophyll cells of apical leaves (d). In Uk-4 ecotype, at this time, high viral accumulation is shown in the stem (e) and mesophyll cells of apical leaves (f). Bars: 100 μm.

Viral movement from the inoculated leaves to the vascular system and then into apical tissues was followed using electron microscopy (Figure 2). Accumulation of TMV-U1 particles in mesophyll cells of Col-0 inoculated leaves was evident at 10 dpi (Figure 2a); however, virions were not observed in the stem, petioles or mesophyll cells of apical leaves at this time point (Figure 2b, c, d). In contrast, a large number of virions were observed in the stem and mesophyll of apical leaves of the Uk-4 ecotype at 10 dpi (Figure 2e and 2f).

**Figure 3:**
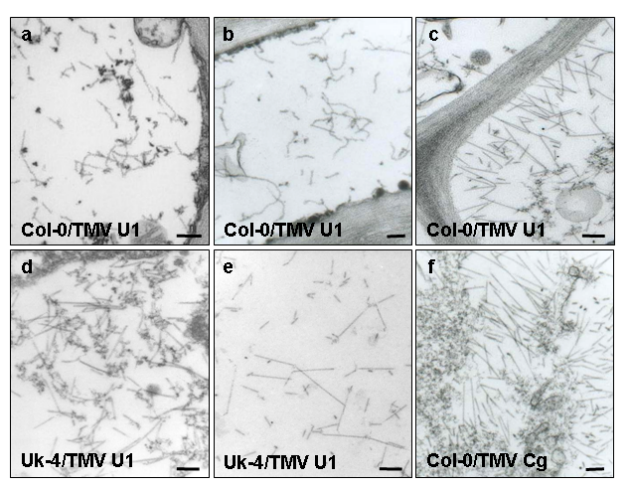
**Curved TMV-U1 virions accumulate in the vascular tissues of infected Col-0 plants**. Electron microscopy images of virus infected tissues in Col-0 and Uk-4 plants were taken at 18 dpi. Curved virions are evident in the vascular tissue of the stem (a) and petiole of apical leaves (b), but normal rigid rod virions appear in the mesophyll of apical leaves (c). Rigid rod virions appear in the stem (d) and petiole of apical leaves (e) in Uk-4 plants. An image of Col-0 vascular cells infected with the TMV-Cg strain was included as a control (f). Bars: 100 μm.

Since the virus is detected in Col-0 apical tissues only after 18 dpi (Figure 1c) [16], cross sections of the vascular zone of rosette leaves, stems and apical leaves were studied at this time point. Surprisingly, curved virions, similar in length to the rigid viral rods (300 nm) were observed in the vascular tissue of the stem (Figure 3a) and in apical leaf petioles (3b). Similar structures were observed in the stems of Col-0 plants in three independent experiments. These could correspond to defectively assembled virions or different associations of viral proteins. However, in the mesophyll cells of apical leaves, normal rigid rods of TMV-U1 were observed (Figure 3c). On the other hand, in the case of Uk-4 infected plants, the stem (3d) and the apical leaf petioles (3e) harboured rigid viral rods. To evaluate if the curved virions appeared when different *Tobamoviruses *infect Col-0 plants, the crucifer infecting TMV-Cg was tested. TMV-Cg efficiently infects several *Arabidopsis *ecotypes and in this case normal shaped virions were always found in the vascular tissue of rosette leaves, stems and apical leaves (Figure 3f).

To investigate if the curved particles were still infective, crude extracts of stem tissues from TMV-U1 infected Col-0 plants were used to inoculate tobacco *Nicotiana tabacum *plants. Local necrotic lesions developed *in tobaco *plants carrying the TMV-resistant *N *gene while systemic infection occurred in sensitive plants lacking the *N *gene, indicating that these particles were still infectious (data not shown).

### The systemic TMV-U1 movement delay trait in the Col-0 ecotype is recessive and nuclear

To determine the genetic basis of the delayed movement of TMV-U1 virions in the Col-0 ecotype, crosses were performed between Col-0 and Uk-4 plants. From the 132 molecular markers used (CAPS and SSLP), 45 were found to be polymorphic (Table 1). Among them, four belonged to chromosome I, 25 to chromosome II, six to chromosome III, four to chromosome IV, and seven to chromosome V (Table 1).

**Table 1:** Polymorphic markers in Col-0 and Uk-4 ecotypes used to map the *DSTM1 *locus

***Marker***	***Chr***	***Type***	***Enzyme***	***Primer Forward (5'→3')***	***Primer Reverse (5'→3')***	***Reference***
m305	I	CAPS	HaeIII	TGAAGCTAATATGCACAGGAG	TTCTCCAGACCACATGATTAT	TAIR
g11447	I	CAPS	EcoRV	CAGTGTGTATCAAAGCACCA	GTGACAGACTTGCTCCTACTG	TAIR
nga59	I	SSLP	-	TTAATACATTAGCCCAGACCCG	GCATCTGTGTTCACTCGCC	TAIR
nga280	I	SSLP	-	GGCTCCATAAAAAGTGCACC	CTGATCTCACGGACAATAGTGC	TAIR
T27E13	II	CAPS	NlaIII	GGAATCATCGCTTGACGACTCC	GGACTTTTCGCCGTGAAGTCG	BAR
ML	II	CAPS	SnabI	CGGAAACACGAAGCTGATGAGTTGGG	CGAGAACAAAATGTGTACGGTGTG	TAIR
T9D9	II	CAPS	MboI	GGTCTCTTTGTCGCAACACTCC	CATGAATGTTGTTTCCAAGTATCC	TAIR
ER	II	CAPS	DdeI	GAGTTTATTCTGTGCCAAGTCCCTG	CTAATGTAGTGATCTGCGAGGTAATC	TAIR
GBF3	II	CAPS	HindIII	AGCGAAGAAAATCACCCAAAGACAGACTC	CTCATTTCATTCTGTTTCTCCGTCAAGTG	TAIR
Ks450	II	CAPS	HpaII	CGGTAGCCGATCCTGATTTGATCAG	TTCCTTATCTCCTTGTCTAACTTCC	Muramoto et al. (1999)
nmF	II	CAPS	BstXI	GGTTTATCACCAAACCAGTTTATTG	TTGTTTGTTCGGGTCTCTCC	Matsumoto & Okada (2001)
nmG	II	CAPS	EcoRV	GGACAGGTAAGAGACAGTAG	AGAAGAGGAGCGTGTATGCT	Matsumoto & Okada (2001)
Ve017	II	CAPS	PstI	GAGCAATCCAGTAGAGGATA	CTTGAAGCTTAAATCTCAGC	TAIR
11444	II	CAPS	EcoRV	CCGGTTTTCGTTCCTGTGTA	CTAAGATCAACCACTGTCCTAGC	BAR
EMB1187	II	CAPS	PstI	AACCTCAGCTGTGGGTTGAT	CATCATTCAGCATGAAGTTTCC	BAR
11697	II	CAPS	HpaII	ATGGGAAGCAAAGCTATTGTATCAG	AACGTTGGAGAGAGACGACA	BAR
F15K20.13	II	CAPS	EcoRI	CACTTCTTCATCGCCGTCTG	CACCTCCTCCACCTCCAGAT	BAR
ATIPT2	II	CAPS	EcoRV	TTTAAGAATTTTGAAGTCCTATACGTT	TCAAGAAACTGTTGTGACTGGA	BAR
F24D13.7	II	CAPS	DraI	GAACTCATTGCCAAAGACGA	TCACAGTGAACCCTCTTCCTG	BAR
T17D12.4	II	CAPS	EcoRV	CAACCACTTATTTGGTACAAGGTG	CTTTTCCAAAGTGCTTACCACA	BAR
12185	II	CAPS	HindIII	CTCGTGGGTTGCGAAAAG	CAGAGCTCTTCATCTTCATCTTCA	BAR
nga1145	II	SSLP	-	GCACATACCCACAACCAGAA	CCTTCACATCCAAAACCCAC	TAIR
nga168	II	SSLP	-	GAGGACATGTATAGGAGCCTCG	TCGTCTACTGCACTGCCG	TAIR
ciw3	II	SSLP	-	GAAACTCAATGAAATCCACTT	TGAACTTGTTGTGAGCTTTGA	TAIR
PLS3	II	SSLP	-	TAGTCGTTTCTCTGGTTGTAG	TTGCCTGTCGATGTAGATTTGT	TAIR
PLS7	II	SSLP	-	GATGAATCTTCTCGTCCAAAAT	GACAAACTAAACAACATCCTTCTT	TAIR
PLS9	II	SSLP	-	GAAATTACGCCGAAAGGTC	CGTCACGAGAGGCACATC	TAIR
CZSOD2	II	SSLP	-	GAATCTCAATATGTGTCAAC	GCATTACTCCGGTGTCGTC	TAIR
C4H	II	SSLP	-	GTTCATGGACGGATGTGTATGC	CTAGTGGTGGTTAAAATATACGCG	TAIR
g4711	III	CAPS	HindIII	CCTGTGAAAAACGACGTGCAGTTTC	ACCAAATCTTCGTGGGGCTCAGCAG	TAIR
Arlim15.1	III	CAPS	EcoRI	TGCTGCTTTATTTTGTCGCGATGTT	GCCAGTTTTTTCCTGCACATCAATC	TAIR
GAPC	III	CAPS	EcoRV	ACGGAAAGACATTCCAGTC	CTGTTATCGTTAGGATTCGG	TAIR
Nga172	III	SSLP	-	CATCCGAATGCCATTGTTC	AGCTGCTTCCTTATAGCGTCC	TAIR
nga112	III	SSLP	-	CTCTCCACCTCCTCCAGTACC	TAATCACGTGTATGCAGCTGC	TAIR
nga12	III IV	SSLP	-	TGATGCTCTCTGAAACAAGAGC	AATGTTGTCCTCCCCTCCTC	TAIR
AG	IV	CAPS	XbaI	CAAACACCATTTAATCTTGACA	CAACAGGTTTCTTCTTCTTCTC	TAIR
g8300	IV	CAPS	HindIII	GGCGGCACTGGTGGTGTAGG	GTTGTCCCCTGTTAAAAGGAGCC	TAIR
nga1139	IV	SSLP	-	TAGCCGGATGAGTTGGACC	TTTTTCCTTGTGTTGCATTCC	TAIR
EG7F2	V	CAPS	XbaI	GCATAGAATTTGACGATAACGAGC	GATCTGTGTAGGACTACGAGAC	TAIR
PLC1	V	CAPS	HindIII	GAATCAGCCATTATCGCATTAC	GGGAACTCAAACTCCTTGTCC	TAIR
nga158	V	SSLP	-	ACCTGAACCATCCTCCGTC	TCATTTTGGCCGACTTAGC	TAIR
nga129	V	SSLP	-	CACACTGAAGATGGTCTTGAGG	TCAGGAGGAACTAAAGTGAGGG	TAIR
nga151	V	SSLP	-	CAGTCTAAAAGCGAGAGTATGATG	GTTTTGGGAAGTTTTGCTGG	TAIR
AThPhyC	V	SSLP	-	CTCAGAGAATTCCCAGAAAAATCT	AAACTCGAGAGTTTTGTCTAGATC	TAIR
ciw10	V	SSLP	-	CCACATTTTCCTTCTTTCATA	CAACATTTAGCAAATCAACTT	TAIR

**Figure 4:**
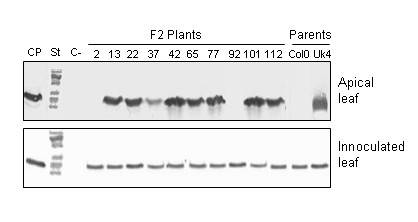
**Systemic movement of TMV-U1 in the F2 population**. Analysis of systemic movement by Western blot in several plants of the F2 population is exemplified. The TMV-U1 coat protein was detected in the inoculated rosette leaves of all plants analysed and in apical leaves of most plants. This analysis was carried out for each of the F2 plants that appear in Table 2. CP: coat protein, St: molecular weight standard, C^-^: negative control (non-inoculated leaves).

To confirm the crosses between Col-0 and Uk-4, polymorphic molecular markers for the five chromosomes were tested in plants from the F_1 _population. All F_1 _tested plants resulted heterozygous, confirming that the crosses were effective (not shown). Sixty plants belonging to the F_1 _[(♀)Uk-4 × (♂)Col-0] population and 50 plants obtained by the reciprocal crossing [(♀)Col-0 × (♂)Uk-4] were used to screen viral movement. Three rosette leaves of each plant were inoculated with TMV-U1, and samples from both apical and inoculated leaves were taken at 12 dpi. These tissues were analyzed by Western blot to detect TMV-U1 CP presence. All F_1 _infected plants accumulated TMV-U1 CP in the apical leaves at 12 dpi, indicating that the susceptible phenotype (Uk-4) is dominant over the "delay in systemic movement" phenotype. These F_1 _plants were then self-pollinated, and 277 plants of the F_2 _population were evaluated for TMV-U1 movement. As an example, analysis of ten F_2_population plants is shown in Figure 4. Accumulation of TMV-U1 CP was examined in the inoculated rosette leaves and in the systemic apical leaves. 198 plants became systemically infected at 12 dpi, while 79 showed delayed systemic movement similar to that of the Col-0 parental ecotype (Table 2). A subsequent analysis of 112 F_2 _plants originating from reciprocal crosses revealed that 82 were systemically infected at 12 dpi and 30 showed a delayed infection (Table 2). These results indicate that the delay in systemic TMV-U1 movement is a recessive trait, probably controlled by a single, monogenic and nuclear locus. This locus was named *DSTM1 *for Delayed Systemic Tobamovirus Movement 1.

**Table 2:** Genetic analysis of the delay in systemic movement of TMV-U1 in Col-0

Cross^a^	Fast movement	Delayed movement	Total	χ^2c^
F1 Uk-4 × Col-0^b^	60	0	60	-
F1 Col-0 × Uk-4	50	0	50	-
F2 Uk-4 × Col-0	198	79	277	1.8
F2 Col-0 × Uk-4	82	30	112	0.76

a: The female parent is represented first in the cross. Six week-old F1 and F2 plants were inoculated and the fast or delayed movement phenotype was determined in infected plants by means of Western blot at 12 dpi.

b: The presented data corresponds to one pair of reciprocal crosses.

c: The values of χ^2 ^and p were calculated to test the probability that this data collected in the segregated population corresponds to the expected values for segregation of 3:1 for a single recessive locus that is responsible of delay in systemic movement of TMV-U1 in Col-0. In both cases p > 0.05.

### Mapping DSTM1

**Figure 5:**
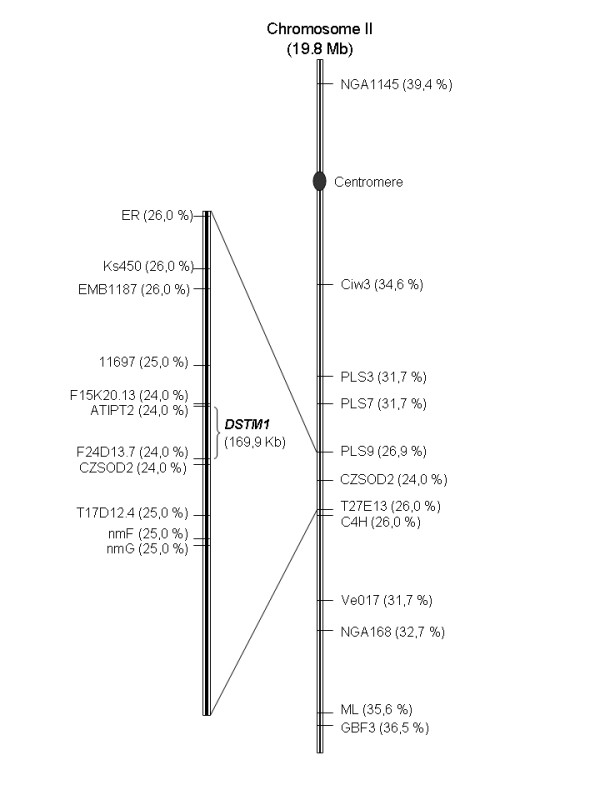
**Location of *DSTM1 *on chromosome II**. Chromosome II map showing SSLP and CAPS molecular markers and the recombination percentages observed in mapping the trait of delay in systemic TMV-U1 movement. An enlarged view of the *DSTM1 *region is shown on the left.

For genetic mapping analysis of the *DSTM1 *locus, F_2 _progeny plants from the UK-4 × Col-0 cross were used. Fifty two TMV-U1 resistant individuals selected for absence of CP in non-inoculated upper leaves at 12 dpi were screened. Co-segregation of this character with different polymorphic molecular markers was studied for each of the five *A. thaliana *chromosomes. In an initial analysis, using four or five polymorphic markers for each chromosome, the lowest recombination percentages obtained were: chromosome I 51.4% with nga 280, chromosome II 36.5% with nga 168, chromosome III 51.4% with nga 172, chromosome IV 50% with AG and chromosome V 56.8% with nga 129. Recombination frequencies between *DSTM1 *locus and the markers on chromosome II were less than 50%, strongly suggesting that *DSTM1 *is located in this chromosome. For further mapping analysis, 21 additional chromosome II CAPS markers were used. Based on recombination frequencies between *DSTM1 *locus and the 25 markers, the *DSTM1 *locus was mapped on the long arm of chromosome II, between ATIPT2 and F24D13.7 CAPS markers. These two markers, at a distance of 169,926 bp from each other, showed the lowest recombination frequency (24%). The location and recombination percentages of all markers are shown in an AGI (bp) map (Figure 5).

## Discussion and conclusion

Systemic movement of TMV-U1 was studied in the *Arabidopsis *Uk-4 and Col-0 ecotypes presenting different rates of systemic infection. The delay in TMV-U1 systemic infection observed in Col-0 occurs at the long-distance movement stage, since CP accumulation in inoculated leaves was similar in both ecotypes (Figure 1). Systemic viral movement delay is not associated with a restriction given by a hypersensitive response, since necrotic lesions are not observed in the Col-0 ecotype. In previous investigations, the expression of resistance markers such as PR-1 had not been detected in *Arabidopsis *infected by TMV-U1 [27,16]. Therefore, this movement restriction indicates a passive response of the plant to the pathogen.

A detailed study of viral infection progress in different plant tissues using electron microscopy revealed that curved virions were present in the vascular tissues of petioles from inoculated leaves and stems of Col-0 plants. However normal rigid rod particles were present in the mesophyll cells of apical leaves, identical to those visualised in the mesophyll cells of inoculated leaves. These abnormal structures occur in a strain-specific manner, since the crucifer infecting TMV-Cg strain, which moves easily through the Col-0 plants (as in all ecotypes tested, [16]) always presented normal rigid rods in the stems of infected plants. This irregular virion shape may be related to the delayed movement in Col-0 plants. Viral entry and exit from phloem elements are the most critical points in systemic virus movement [1]. Inside phloem cells, protein synthesis or replication does not occur. Prior works have suggested that entry and exit from the vascular tissues could require a different set of host factors than those acting in the other infected tissues [1]. Thus, the vascular zone of Col-0 ecotypes could, for instance, interfere with correct viral assembly, stability or entry into the vascular tissue from the infected leaf. Nevertheless, when the virus exits the phloem and enters the mesophyll cells in systemic tissues, its structure assembles correctly again. Moreover, we demonstrated that the curved virions isolated from infected Col-0 plants were infective.

The phenotypic differences observed between the Uk-4 and Col-0 ecotypes prompted us to look for a host factor which could be related to this delay of systemic virion movement. Genetic crosses demonstrated that this delay is a recessive trait, since the rate of systemic movement was normal in all F_1 _plants, and is probably controlled by a monogenic nuclear locus. This locus was named *DSTM1 *for Delayed Systemic Tobamovirus Movement 1 and was mapped on the long arm of chromosome II, between sections 11,832,469 – 12,002,395 bp of *Arabidopsis *Col-0, using molecular markers. According to the *Arabidopsis *genome database (TAIR; ) this region contains 43 loci and most of the genes within this region are unknown or putatively assigned. Among these unknown genes, four are related to transport function, one is involved in cell wall biosynthesis (a gluconyl transferase) and another encodes a protein with fucosidase activity. All of these functions are related to viral movement [28]. Whether if any of these loci is functionally responsible for the delayed viral movement phenotype, for instance interfering with the entrance into the vascular system, remains to be determined. Alternatively, the locus could participate in the stability or correct assembly of viral particles in the vascular tissue. This type of restriction leads to a passive resistance [29], because viral movement is a consequence of the absence of a host factor.

Other loci affecting systemic viral movement in a dominant or semi-dominant way in *Arabidopsis thaliana *have been identified and characterised. As in the present study, most are specific to one particular virus, since the infective process of the related Tobamovirus TMV-Cg is not affected in Col-0. Several other loci are related to restrictions in systemic movement [30]. For example, the *VSM1 *locus, although dominant, restricts *Turnip vein clearing tobamovirus *(TVCV) systemic movement at the level of phloem entry or exit [31]. In the Col-0 ecotype, two loci *RTM1 *and *RTM2 *have been proven to be responsible for restriction of *Tobacco etch virus *systemic movement. These loci were mapped to chromosomes I and V respectively and their products were localised in the phloem [14,15,32]. However neither direct nor indirect interactions between these proteins and virions have been found. Higher resolution mapping of *DMST1 *will lead to precise identification of the gene responsible for the delay in systemic TMV-U1 movement. This undoubtedly will contribute to the understanding of the complex interactions between virus and hosts leading to efficient infections, and therefore, for understanding resistance development.

## Authors' contributions

CS participated in the design of the study, carried out the electron microscopy, hybridisation assays, genetic crosses and genetic marker analysis. JGC carried out the mapping on the F_2 _population, genetic marker design and participated in drafting the manuscript. FJ and PM collaborated in phenotypic population studies by immunoblots and mapping. CM conducted plant inoculation and together with JTM, collaborated in drafting the manuscript. PAJ designed the experimental procedure, was involved in revising the manuscript critically for important intellectual content and gave final approval of the version to be published. All authors read and approved the final manuscript.
